# Synergistic anti-tumor activity of acadesine (AICAR) in combination with the anti-CD20 monoclonal antibody rituximab in *in vivo* and *in vitro* models of mantle cell lymphoma

**DOI:** 10.18632/oncotarget.1455

**Published:** 2014-01-25

**Authors:** Arnau Montraveta, Sílvia Xargay-Torrent, Mónica López-Guerra, Laia Rosich, Patricia Pérez-Galán, Itziar Salaverria, Silvia Beà, Susana G. Kalko, Mercè de Frias, Clara Campàs, Gaël Roué, Dolors Colomer

**Affiliations:** ^1^ Experimental Therapeutics in Lymphoid Malignancies Group, Institut d'Investigacions Biomèdiques August Pi i Sunyer (IDIBAPS), Barcelona, Spain; ^2^ Bioinformatics Core Facility, IDIBAPS, Barcelona, Spain; ^3^ Advancell-Advanced In Vitro Cell Technologies S.A., Barcelona, Spain; ^4^ Hematopathology Unit, Hospital Clínic, Barcelona, Spain

**Keywords:** Acadesine, rituximab mantle cell lymphoma, xenograft mouse model

## Abstract

Mantle cell lymphoma (MCL) is considered one of the most challenging lymphoma, with limited responses to current therapies. Acadesine, a nucleoside analogue has shown antitumoral effects in different preclinical cancer models as well as in a recent phase I/II clinical trial conducted in patients with chronic lymphocytic leukemia. Here we observed that acadesine exerted a selective antitumoral activity in the majority of MCL cell lines and primary MCL samples, independently of adverse cytogenetic factors. Moreover, acadesine was highly synergistic, both *in vitro* and *in vivo*, with the anti-CD20 monoclonal antibody rituximab, commonly used in combination therapy for MCL. Gene expression profiling analysis in harvested tumors suggested that acadesine modulates immune response, actin cytoskeleton organization and metal binding, pointing out a substantial impact on metabolic processes by the nucleoside analog. Rituximab also induced changes on metal binding and immune responses. The combination of both drugs enhanced the gene signature corresponding to each single agent, showing an enrichment of genes involved in inflammation, metabolic stress, apoptosis and proliferation. These effects could be important as aberrant apoptotic and proinflammatory pathways play a significant role in the pathogenesis of MCL. In summary, our results suggest that acadesine exerts a cytotoxic effect in MCL in combination with rituximab, by decreasing the proliferative and survival signatures of the disease, thus supporting the clinical examination of this strategy in MCL patients.

## INTRODUCTION

Mantle cell lymphoma (MCL) represents 5-10% of all non-Hodgkin lymphomas (NHLs) and is one of the most aggressive lymphoid neoplasms with poor prognosis. Its genetic hallmark is the chromosomal translocation t(11;14)(q13;q32), which leads to cyclin D1 overexpression with the consequent cell cycle deregulation [1]. MCL cells carry a high number of secondary genetic alterations that increase the oncogenic potential of cyclin D1 and frequently inactivate the cellular response to DNA damage. In addition, other mechanisms such as activation of cell survival pathways are integrated to drive MCL pathogenesis. Current frontline combination chemotherapies and intensive chemoimmunotherapy followed by stem-cell transplantation have improved the outcome for patients with this disease [2]. Although these regimens have high initial response rates, most patients relapse and die from disease-related complications [1]. The introduction of rituximab, a chimeric mouse anti-human CD20 monoclonal antibody, has shown improvement of response rates when used in combination with standard chemotherapy [3;4]. In the last years, new strategies that target crucial biological pathways such as ubiquitin-proteasome system, mTOR pathway and BCR signaling have been developed [2;5;6]. In particular, recently it has been described that Ibrutinib, a BTK inhibitor shows durable single-agent efficacy in relapsed or refractory MCL [7]. Acadesine (5-aminoimidazole-4-carboxamide-1-D-ribofuranoside, AICA-riboside or AICAR) is a nucleoside analogue initially developed as a cardioprotective agent, with a different mechanism of action compared to standard nucleoside analogues, like fludarabine [8]. When added to cell cultures or administered to animals or humans, acadesine is phosphorylated to AICA-ribotide (ZMP), the natural endogenous intermediate in the *de novo* purine nucleotide biosynthesis, which can function as an AMP mimic and activate AMP-activated kinase (AMPK), a protein that regulates the responses of the cell to energy changes [9]. Although acadesine is commonly used as an AMPK activator, there are compelling evidences that acadesine anti tumoral effects could be mediated, at least in part, independently of the AMPK pathway [10-13]. Nevertheless, at present, the exact nature of the AMPK-independent effects of acadesine in leukemic cells is not clearly understood. Many studies have shown that acadesine can inhibit proliferation, and induce apoptosis in multiple myeloma [14], neuroblastoma [15], glioblastoma [16], childhood acute lymphoblastic leukemia (ALL) [17], colon cancer [18], and breast and prostate cancer cell lines [19]. In particular, acadesine exerts a pro-apoptotic activity in a wide range of B lymphoid malignancies [20], being cells from chronic lymphocytic leukemia (CLL) the most sensitive to this agent [13;21]. Recently, a phase I/II clinical trial conducted in relapsed/refractory CLL patients has demonstrated a remarkable activity of the drug in the clinical settings [22].

In this study, we show that acadesine exerts a specific antitumoral activity in the majority of MCL cell lines and primary samples, and synergizes with rituximab both *in vitro* and *in vivo*. Furthermore, we provide gene expression profiling (GEP) data on the putative mechanisms of action of acadesine-rituximab combination, supporting clinical examination of this strategy in MCL patients.

## RESULTS

### Acadesine exerts a cytotoxic effect both in MCL cell lines and MCL primary samples

**Figure 1:**
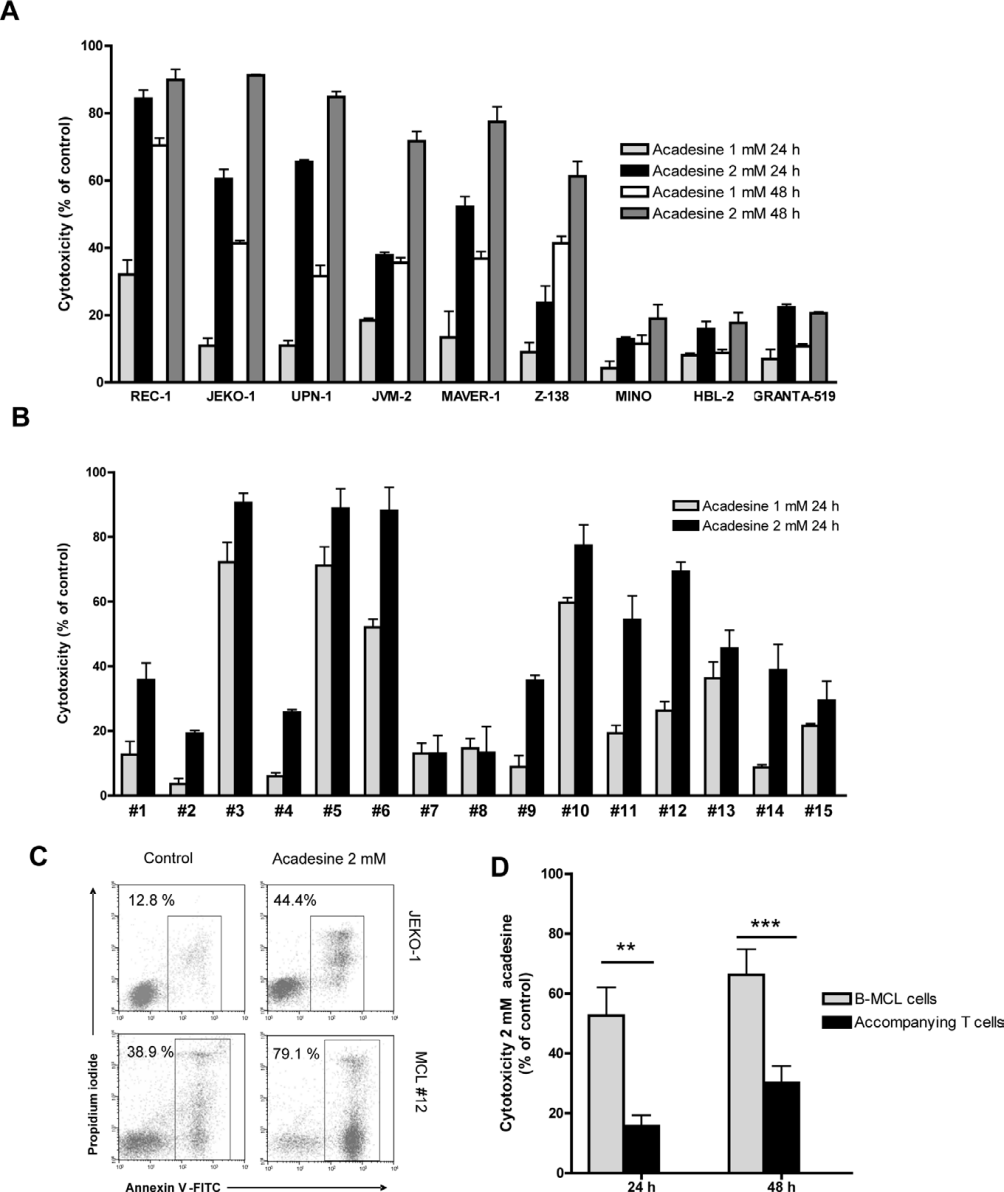
Acadesine induces cytotoxicity in both MCL cell lines and MCL primary samples A. MCL cell lines were incubated with acadesine 1 mM and 2 mM for 24 and 48 hours and cytotoxicity was measured by Annexin V labeling. Data show the mean ± SEM of three independent experiments. B. Primary MCL cells were incubated with acadesine 1 mM and 2 mM for 24 hours and cytotoxicity was measured as above. Data show the mean ± SEM of three replicates. C. Representative flow cytometric plots of Annexin V/Propidium iodide labeling in a representative MCL cell line (JEKO-1) and a primary MCL sample (MCL#12) treated with acadesine 2 mM for 24 hours. D. Acadesine cytotoxicity in B tumoral and T normal lymphocytes from MCL cases. Results show the mean cytotoxicity of 10 primary MCL samples ± SEM analyzed after incubation with acadesine 2 mM for 24 hours. (** *P<* 0.01, *** *P<* 0.001)

Nine MCL cell lines were exposed for 24 or 48 hours to increasing doses of acadesine ranging from 0.1 to 2 mM, and IC_50_ and LD_50_ values were calculated from data obtained by the MTT and the Annexin V assays, respectively. As shown on Table 1, most of the cell lines analyzed (REC-1, JEKO-1, UPN-1, JVM-2, MAVER-1 and Z-138) showed a IC_50_ lower than 1 mM after 48 hours of acadesine incubation. Three cell lines (MINO, HBL-2 and GRANTA-519), showed a IC_50_ higher than 2 mM, the maximum dose tested. The analysis of the LD_50_ values followed the same pattern, being REC-1, JEKO-1 and UPN-1 the most sensitive cell lines to acadesine-induced cell death, with LD_50_ below 1 mM after 48 hours of incubation (Figure 1A and 1C). Z-138, JVM-2 and MAVER-1 cell lines showed LD_50_ around 1.5 mM at 48 hours and, accordingly to the MTT assay results, MINO, HBL-2 and GRANTA-519 exhibited low sensitivity to acadesine with a not-reachable LD_50_. Acadesine-evoked cell death increased in a time- and a dose-dependent manner, as illustrated in Figure 1A. Deletion at 17p, affecting *TP53* or the total amount of copy number alterations (CNA), including trisomies or monosomies that carried most of the MCL cell lines (Table 1) did not affect the susceptibility of MCL cells to acadesine.

TABLE 1Genetic characteristics of MCL cel lines and MCL primary samples

**Table:** 

MCL cell lines	Copy number alterations^a^			Genetic alterations	Acadesine sensitivity 48 h (mM)		Combination index
	Total	Trisomies	Monosomies	*TP53^b^*	IC_50_	LD_50_	Acadesine-Rituximab^d^
REC-1	37	0	0	wt/mut	0.28	0.65	0.822
JEKO-1	79	4	0	del/mut	0.59	1.00	0.400
UPN-1	47	1	0	del/mut	0.64	0.78	0.786
JVM-2	8	0	0	wt	0.98	1.51	0.918
MAVER-1	47	0	0	del/mut	0.50	1.26	1.119
Z-138	25	3	0	wt	0.14	1.46	0.646
MINO	22	3	0	UPD/mut	NR	NR	0.515
HBL-2	65	0	0	del/mut	NR	NR	0.813
GRANTA-519	30	1	0	del/wt	NR	NR	1.154

**Table:** 

MCL samples (% tumoral cells)	Copy number alterations^a^			Genetic alterations	% Acadesine cytotoxicity 24h		Combination index
	Total	Trisomies	Monosomies	*TP53^b^*	1 mM	2 mM	Acadesine-Rituximab^d^
MCL #1 (83%)	41	1	0	del/wt	12.68	35.75	-
MCL #2 (85%)	9	0	1	wt	3.58	19.22	-
MCL #3 (97%)	0	0	0	wt	72.20	90.50	0.551
MCL #4 (89%)	2	0	0	wt	6.05	25.71	-
MCL #5 (91%)	25	0	0	del/wt	71.10	88.80	0.715
MCL #6 (86%)	13	1	0	del/mut	52.04	88.71	-
MCL #7 (86%)	14	0	0	wt	12.97	13.01	-
MCL #8 (80%)	4	0	0	mut	14.64	13.25	-
MCL #9 (77%)	2	0	0	del/wt	8.87	35.51	-
MCL #10 (84%)	1	0	0	del/wt	59.70	77.30	-
MCL #11 (77%)	0	0	0	wt	19.31	54.30	-
MCL #12 (76%)	15	0	0	wt	26.22	69.21	-
MCL #13 (97%)	4	0	0	del/^c^	36.25	45.48	0.575
MCL #14 (94%)	19	0	1	wt	8.70	38.81	0.681
MCL #15 (92%)	8	0	0	UPD/^c^	21.59	29.35	0.462

Abbreviations: del, deletion ; mut, mutation; wt, wild type; UPD, uniparental disomy; NR, not reached; ND, not determined

a del and UPD detected by SNP-100K array

b *TP53* mutational status detected by direct sequencing

c mutations not analyzed

d Doses used: JEKO-1, acadesine 1 mM and rituximab 1 μg/ml; other cell lines and primary samples, acadesine 1 mM and rituximab 40 μg/ml

Then, isolated tumor cells from 15 MCL samples were exposed for 24 hours to acadesine 1 and 2 mM, and cell viability was analyzed by annexin V labeling. As shown on Table 1 and illustrated on figure 1C, similarly to what observed in MCL cell lines, acadesine also induced apoptosis in primary patient cells, even though this effect was heterogeneous among our set of MCL primary cultures (Figure 1B). Six out of fifteen cases (40%) showed a response above 25 % to 1 mM acadesine, while 12 of 15 cases (80%) achieved these responses at 2 mM acadesine, being the mean cytotoxicity at this dose 48.28 ± 27.97%. Again, no association could be observed between the response to acadesine and the presence of *TP53* anomalies and CNAs in the set of primary MCL samples studied. Despite all of them harbored a high percentage of tumoral B-cells (range 76-97%) (Table 1), we analyzed the activity of acadesine in B-tumoral and the accompanying T-cells in 10 out of the 15 MCL cases studied. Using a triple CD19/CD3/Annexin V labeling, we found that B tumor cells (CD19+) were significantly more sensitive to a 2 mM dose of the drug than the normal T-cell subset, both at 24 and 48 hours (Figure 1D, *P*<0.01 at 24 hours and *P*<0.001 at 48 hours).

Altogether, these results suggest that acadesine is active in the majority of MCL cell lines and primary samples, where it exerts a selective antitumoral effect, regardless of genetic alterations and adverse prognostic factors.

### Acadesine and rituximab exert a synergistic cytotoxic effect

**Figure 2:**
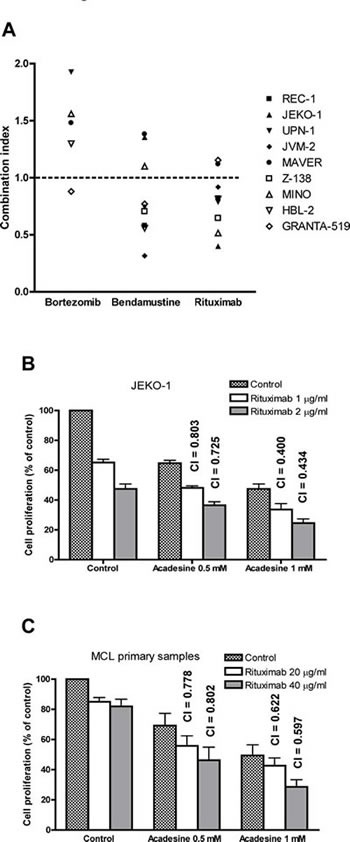
Acadesine shows a synergistic effect with rituximab *in vitro* and *in vivo* A. Acadesine was combined with bortezomib, bendamustine and rituximab in a panel of 9 MCL lines and inhibition of proliferation was analyzed by the MTT assay. The graph shows the CI of the combination of acadesine 1 mM with bortezomib 5 nM, bendamustine 25 μM, and rituximab 40 μg/ml (1 μg/ml for JEKO-1) for each cell line. The combinations with CI > 2 are not shown in the graph. B. Combination of acadesine with rituximab in JEKO-1 cell line. Cells were pre-incubated for 24 hours with acadesine, followed by a 24-hour exposure to rituximab. Inhibition of proliferation was analyzed by the MTT assay. Data show the results of three independent experiments ± SEM and CI are indicated above the bars. C. Combination of acadesine with rituximab in MCL primary samples. Primary cells were incubated with acadesine and rituximab as above. Data show the mean of 5 samples ± SEM and CI are indicated above the bars.

We further investigated potential interactions of acadesine with drugs currently approved for the treatment of relapsed/refractory MCL, including bortezomib, bendamustine and rituximab. For this aim, a panel of MCL cell lines were incubated for 48 hours with two different doses of acadesine (0.5 and 1 mM), bortezomib (2.5 and 5 nM) and bendamustine (25 and 50 μM). Rituximab experiments were performed after incubation of cells for 24 h with acadesine, followed by an additional 24 h incubation with or without two different concentrations of rituximab (20 and 40 μg/mL), except for JEKO-1 cells where rituximab was used at 1 and 2 μg/ml. Inhibition of proliferation was measured using the MTT assay. Then the combination index (CI) using the Chou and Talalay method were evaluated for each drug combination and represented in Figure 2A. An antagonistic effect was observed when acadesine was combined with 5 nM bortezomib. When used in combination with bendamustine 25 μM, acadesine displayed either additive or synergistic cytotoxic activity, depending on the MCL cell line, and being the cell lines carrying a P53 wild type phenotype those with the higher synergistic effect between these two drugs. Interestingly, a synergistic effect of acadesine plus rituximab was observed in 7 out of the 9 MCL cell lines tested, with CI values ranging from 0.400 to 0.918, with no correlation with any known MCL genetic alteration (Table 1). The two remaining MCL cell lines (MAVER-1 and GRANTA-519), showed CI values closed to 1, indicative of an additive or a slightly antagonistic effect.

In 5 MCL primary samples, the combination of acadesine with rituximab was also synergistic at all the concentrations tested (Table 1), being the best drug interaction obtained with acadesine 1 mM and rituximab 40 μg/ml (mean CI = 0.597 ± 0.102, Figure 2C). Importantly, the synergistic effect observed in primary MCL cells was independent of the initial response to acadesine, being rituximab able to sensitize MCL cells and to overcome their resistance to the nucleoside analog.

To validate the specificity of the cooperation between acadesine and rituximab in MCL, we evaluated the cytotoxic effect of both drugs either alone or in combination in two CLL cell lines, namely MEC-1 and MEC-2, and in a set of 4 primary CLL cultures. We observed that in CLL cells the acadesine-combination lacked the synergistic activity observed in MCL cells, with CI values ranging from 1.181 to 8.336, being additive in the MEC-1 cell line and antagonistic in the MEC-2 cell line, as well as in all the CLL primary samples tested (data not shown).

Altogether, these results suggest that among the standard agents currently used in the clinical, rituximab presents the best combinational activity with acadesine, and that this effect may be specific for the MCL model.

### The combination of acadesine and rituximab inhibits tumor outgrowth in a mouse xenograft model of MCL

**Figure 3:**
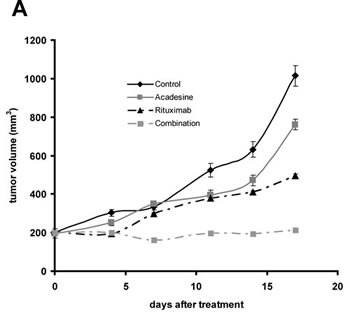
Acadesine-rituximab combination inhibits MCL tumor outgrowth A. SCID mice were inoculated subcutaneously with JEKO-1 cells and treated with acadesine (400 mg/kg 5 days a week), rituximab (10 mg/kg once a week) or both drugs. Tumor growth is represented as the mean ± SEM (n=6) (** *P*< 0.01, *** *P*< 0.001)

To validate the synergism between acadesine and rituximab observed in JEKO-1, we evaluated the antitumoral effect of this combination in a CB17-SCID mouse xenograft model of MCL generated using this cell line. Animals were randomly assigned into different groups with 6 mice per cohort and treated with acadesine at dose level of 400 mg/kg body weight five days a week, rituximab at 10 mg/kg body weight once a week, both drugs as above or the equivalent volume of vehicle. When compared to vehicle-treated mice, after 18 days of treatment, we observed that tumor burden was significantly reduced in mice treated either with acadesine (*P*<0.01) or with rituximab (*P*<0.001) as single agents. We observed a reduction in tumor size of 31.22 ± 15.68% in acadesine-receiving animals and of 63.85 ± 10.88% in the rituximab arm. The combination of both agents was significantly more effective than the two drugs alone (*P*<0.001 compared to acadesine, *P*<0.01 respect to rituximab) leading to an almost complete inhibition of tumor growth (Figure 3A). Mice tolerated well the treatment, and no effect on animal vital parameters was reported, in any of the treatment arms. Thus, these results suggest that the combination of acadesine with rituximab might represent a safe and efficient therapeutic approach for the treatment of MCL tumors.

### The combination of acadesine and rituximab modulates genes related to inflammation, metabolic stress, proliferation and survival pathways in MCL

**Figure 4:**
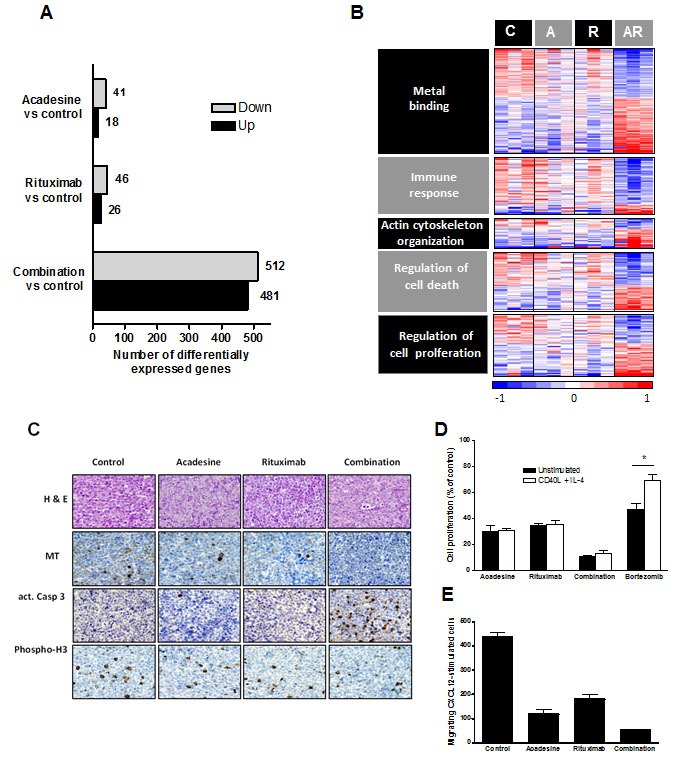
Gene expression profile analysis of mice-treated tumors GEP analysis of tumors was performed and graph shows the number of differentially expressed genes from each treatment compared to the control with a FDR below 0.2 and an absolute FC above 1.4 B. Heatmap displaying genes modulated by the combination clustered according to GO terms. Relative gene expression levels are color-coded as indicated at the bottom. C: control; A: acadesine; R: rituximab; AR: combination. C. Immunostaining of harvested tumors of mouse xenograft MCL after acadesine, rituximab and acadesine-rituximab treatment. Histological sections from representative tumors of each treatment stained with hematoxylin and eosin and specific antibodies against metallothionein, activated caspase-3 and phospho-histone 3 (x40) D. JEKO-1 cells were treated with acadesine, rituximab and the combination after a CD40L + IL-4 stimulation and inhibition of proliferation was measured by MTT assay. Data show the mean of three independent experiments ± SEM. E. Migration assay of JEKO-1 cells treated with acadesine, rituximab and the combination. Graph shows the number of CXCL12-stimulated cells migrating after each treatment.

In order to understand the basis of the synergy between acadesine and rituximab, we next examined changes in gene expression profiles in a set of representative harvested tumors from each cohort (n=3 tumors/group). We determined the differentially expressed genes from each treatment compared to the control with a false discovery rate (FDR) below 0.2 and an absolute fold change (FC) above 1.4 using the Rank Products method in the TM4-MEV platform. Treating the cells with acadesine was associated with the lowest number of gene modulations (18 genes up-regulated and 41 genes down-regulated), whereas after rituximab treatment 26 genes were up-regulated and 46 genes were down-regulated. Acadesine and rituximab treatment affected the expression of a much higher number of genes, being 481 genes up-regulated and 512 genes down-regulated (Figure 4A and Supplemental Table 1). To gain insight into the biological function of the treatment-related differential expression profiles, we conducted a gene onthology (GO) enrichment analysis using the DAVID application. Both acadesine and rituximab monotherapies were found to be related to an enrichment of genes involved in metal binding and immune response, while genes related to actin cytoskeleton organization and immune response, were more specifically regulated by acadesine and rituximab, respectively. Importantly, the treatment with acadesine-rituximab combination allowed to the identification of the same GO terms, but with improved significance (*P*<0.001). Additionally, an enrichment of genes related to regulation of cell death and cell proliferation was detected in tumors receiving this combination (Table 2). Figure 4B showed an overview of the genes modulated by the combination clustered according to the previous described GO terms.

**TABLE 2:** GO terms identified with the DAVID functional anotation

GO terms	Acadesine vs control		Rituximab vs control		Combination vs control	
	Genes	P value	Genes	P value	Genes	P value
**Metal binding**	3	1.7 × 10-3	20	1.3 × 10-2	202	3.3 × 10-7
**Immune response**	8	1.4 × 10-2	7	4.8 × 10-2	78	6.9 × 10-9
**Actin cytoskeleton organization**	4	5.0 × 10-2	-	NS	30	2.2 × 10-5
**Regulation of cell death**	-	NS	-	NS	64	7.2 × 10-4
**Regulation of cell proliferation**	-	NS	-	NS	63	8.6 × 10-4

Then, GSEA was used to identify the gene-sets and pathways modulated by each treatment considering a FDR<0.05 (Table 3). For acadesine, the statistically significant normalized enrichment scores (NES) were achieved by the metallothionein node (NES=-1.92) and the WNT pathway (NES=-1.97). In tumors treated with rituximab, we found also an enrichment of the metallothionein node (NES=-1.86). Furthermore, pathways involved in B/T cell calcium signaling (NES=-1.87), CD40 (NES=-1.89) and NF-κB pathways (NES=-1.91) were also down-regulated. A significant regulation of all these pathways, except for WNT, was also observed in tumors treated with the drug combination. Additionally, we observed down-regulation of genes involved in inflammatory response (NES=-2.02), apoptosis (NES=-1.88), metabolic stress (NES=-1.87), as well as interferon signaling (NES=-2.82) and Toll pathway (NES=-1.83), both of them involved in inflammation (Table 3).

**TABLE 3:** Gene sets regulated after treatment with acadesine, rituximab or the combination

Gene sets	Acadesine vs control			Rituximab vs control			Combination vs control		
	Genes	NES	FDR	Genes	NES	FDR	Genes	NES	FDR
**Interferon pathway**	-		NS	-	-	NS	92	-2.82	<1.0 × 10 ^−4^
**B/T cell calcium signaling**	-	-	NS	41	-1.87	1.2 × 10 ^−2^	54	-2.38	<1.0 × 10 ^−4^
**NFκB** pathway	-	-	NS	70	-1.91	1.2 × 10 ^−2^	66	-2.28	<1.0 × 10 ^−4^
**Inflammatory response**	-	-	NS	-	-	NS	23	-2.02	1.4 × 10 ^−3^
**CD40 pathway**	-	-	NS	62	-1.89	1.2 × 10 ^−2^	49	-1.96	2.6 × 10 ^−3^
**Metallothionein node**	4	-1.92	2.8 × 10 ^−2^	5	-1.86	1.2 × 10 ^−2^	5	-1.96	3.5 × 10 ^−3^
**Antiapoptosis**	-	-	NS		-	NS	8	-1.88	3.7 × 10 ^−3^
**Metabolic stress**	-	-	NS	-	-	NS	5	-1.87	4.3 × 10 ^−3^
**Toll pathway**		-	NS		-	NS	9	-1.83	6.0 × 10 ^−3^
**Wnt pathway**	9	-1.97	2.9 × 10 ^−3^	-	-	NS	-	-	NS

Gene sets were considered significant when FDR<0.05

NES, Normalized Enriched Score; FDR, False discovery rate; NS, not significant

In an attempt to validate the relevance of some of these profiles at the functional level, we first selected to analyze the variation of the metallothionein node, by immunohistochemical detection of metallothionein proteins in the different treatment groups. For this aim, histologic sections from representative whole tumors were labeled with specific antibodies against metallothionein as indicated in material and methods. As shown on figure 4D (panel MT), and in accordance with our GEP results, down-regulation of MT staining was observed in both acadesine- and rituximab-treated groups, that became almost complete in the combination-treated tumors.

Regarding the immune response profile, as the GSEA analysis identified the cytokine receptor CD40 among the main components of this profile, and as it has been reported that CD40 participates in the survival, cell growth, and drug resistance in MCL [23], we performed a co-stimulation assay of JEKO-1 cells with recombinant CD40L + interleukin-4 (IL4), followed by cell exposure to the different drugs and determination of cell viability. As shown on figure 4D, stimulation of MCL cells with CD40L+IL4 did not prevent the decrease in cell proliferation either in acadesine, rituximab-, or the combination, contrasting with what observed when MCL cells were incubated with bortezomib. These results suggest that CD40 pro-survival signaling does not affect significantly the acadesine-rituximab cytotoxic activity in MCL cells.

Next, as the actin cytoskeleton organization node contained genes related to the migration of cells, we performed chemotaxis assays in JEKO-1 cells exposed to acadesine, rituximab and the combination. We observed that both agents were able to inhibit the migration of JEKO-1 cells, and that this effect was higher in cells treated with the combination (Figure 4E).

Finally, and in agreement with our DAVID analysis, showing that the regulation of cell death and proliferation were significantly affected by acadesine-rituximab combination in MCL tumors, by immunohistochemistry we observed a higher expression of the activated form of caspase-3 (figure 4C, panel act.Casp3) and a reduction in the phosphorylation status of the proliferation marker histone H3 (figure 4C, panel Phospho-H3), in the tumor tissues treated with acadesine plus rituximab, when compared to vehicle- and single agent-treated tumors.

In summary, the combination of both acadesine and rituximab enhanced the gene signature corresponding to each single agent, showing an enrichment of genes involved in inflammation, metabolic stress, apoptosis and proliferation. These effects could be important as aberrant apoptotic and proinflammatory pathways play an important role in the pathogenesis of MCL.

## DISCUSSION

Acadesine has demonstrated to efficiently block cell proliferation in several tumor models, in association with decreased fatty acid and protein synthesis [24]. Besides inhibiting cell proliferation, acadesine induces also apoptosis in different tumor cell types [13;15;21;25-27] and autophagy [28]. Recently, the first phase I/II trial has been conducted in relapsed/refractory CLL patients showing a manageable and safe profile and with the conclusion that acadesine may represent a valuable agent for the treatment of this entity [22].

In the present study we show that acadesine exerts a pro-apoptotic effect in the majority of MCL primary cells and cell lines in a time- and dose-dependent manner, at physiologically achievable doses. The LD_50_ values are slightly higher than those observed in CLL cells, which showed a LD_50_ of 380 ± 60 μM [21], but similar to those reported previously in a reduced subset of MCL cases [20]. Interestingly, the maximum acadesine dose tested in our experiments (2 mM), did not induce apoptosis in accompanying T-cells from any of the MCL cases analyzed, thus arguing in favor of the specificity of the drug. The same specificity for tumoral B cells have been described in the CLL model [21], that sharply contrasts with the lack of selectivity of other anti-leukemic drugs, like conventional nucleoside analogs [29]. Our results demonstrate that MCL response to acadesine is independent of *TP53* status and that it is not affected by the occurrence of CNAs. The situation seems to differ from other cell types, as it has been recently described that acadesine elicited a selective apoptotic response in trisomic mouse embryonic fibroblasts [30] and chromosomal instability-driven colorectal cancer cell lines [31].

The clinical course of MCL is characterized by an initial high response rate but a constant relapse pattern, resulting in a poor long-term outcome [2]. It has been reported that first-line chemotherapy including rituximab is associated with significantly improved survival in older patients diagnosed with MCL compared with chemotherapy alone [3]. In the last years, the proteasome inhibitor bortezomib and bendamustine, an hybrid drug between a nucleoside analog and an alkylating agent, have been approved in USA for the treatment of patients with relapsed MCL patients, either alone or in combination with rituximab [2]. Here, we observed an antagonistic effect between bortezomib and acadesine whereas for bendamustine we detected an additive or synergistic effect depending on the MCL cell line. Our results demonstrated that rituximab is the best useful complementary drug to use in combination with acadesine, compared with bendamustine and bortezomib. The efficacy of the acadesine-rituximab combination was independent of *TP53* mutational status in MCL cells, whereas the synergism effect of acadesine plus bendamustine was higher in cell lines with P53 wild type phenotype, consistently with the known role of P53 in MCL cell response to bendamustine [32]. Importantly, this synergistic effect between acadesine and rituximab appeared to be specific for MCL, as it was not observed in CLL cells. The synergistic *in vitro* effect between acadesine and rituximab was then confirmed *in vivo* using a xenograft mouse model, where a remarkable regression of the tumor was observed in animals dosed with the drug combination. The antineoplastic effect of acadesine alone has already been reported on the growth of mice xenograft models of prostatic tumors [19], CML [28] and retinoblastoma [33]. Our *in vivo* results using a xenograft model showed that the treatment with acadesine had a modest anti tumoral activity, suggesting that this drug alone could have limited therapeutic application as a single agent. In contrast, an important anti tumoral activity was observed when combined with rituximab, leading to almost complete inhibition of tumor growth. These results are in agreement with the notion that rituximab significantly improves the response rates in MCL patients when used in combination with standard chemotherapy [2-4], as well as with the last agents approved for the treatment of relapsed/refractory MCL patients, like bortezomib [34], temsirolimus [35], and lenalidomide [36]. Based on our present results, acadesine-rituximab may also warrant potential use in the clinical practice.

To date, there is still no absolute consensus of the *in vivo* mechanism of action of rituximab or rituximab-based combination. Based on *in vitro* studies, rituximab appears to mediate the depletion of B-cells by several mechanisms that are dependent on the host immune system, including complement-dependent and antibody-dependent cellular cytotoxicities [37-39]. It has also been proposed that rituximab might sensitize lymphoma cells to chemotherapy, and have direct antiproliferative and apoptotic effects [38]. Using a GEP approach, we aimed to know the signaling pathways by which acadesine exerts its anti-tumor effect in combination with rituximab. We observed that the number of genes down- and up-regulated after acadesine and rituximab treatments as single agents was low, but that it increased notably with the combination treatment in line with the observed tumor burden inhibition. We found 5 GO terms differentially expressed: metal binding, immune response, actin cytoskeleton organization and regulation of cell death and proliferation. Acadesine alone was able to modulate immune response, actin cytoskeleton organization and metal binding, having a substantial impact of the drug toward metabolic processes, accordingly to a recent microRNA analysis in acadesine-exposed hepatocytes [40]. Rituximab also induced changes on metal binding and immune responses. Accordingly, it has been reported that rituximab was capable of inducing a calcium flux in B-cells through its ability to associate with B-cell receptor (BCR) [41]. CD20 activity is also known to be associated with BCR activation through the induction of Src family tyrosine kinases and the activation of the MAPK pathway [42]. Conversely, rituximab has been shown to inhibit BCR signaling, and different signaling pathways involving MAPK, PI3K/Akt, NF-κB and mTOR kinases [43]. Among the metal binding core of genes, we observed a decrease in genes implicated in response to metal ion and negative regulation of cell growth, namely *MT1G* and *MT1X*, that codify for two members of the metallothionein family, whose expression has been reported to constitute an independent risk factor in diffuse large B cell lymphoma [44]. We confirmed by inmunohistochemistry the downregulation of these metallothionein proteins in tumors from the three treatment arms, but especially in tumors having received the combination of drugs. Similarly, when considering the immune response profile in the combo group by GEP analysis, we observed an accentuated downregulation of the CD40 signaling pathway. It has been described that the microenvironment, including cytokines, has a central role on MCL cell survival and drug resistance, and that the CD40 system acts as a growth-promoting stimulus [23]. Thus, pharmacological interference with this signaling pathway may have therapeutical relevance in MCL, as demonstrated by the use of the CD40-targeting antibody dacetuzumab in combination with rituximab in *in vitro* and *in vivo* models of MCL [45]. Of special relevance, we observed that acadesine, rituximab and their combination were able to overcome the CD40 pro-survival effect in MCL cells, offering a glimpse for further combination therapy in this model. Finally, our results also showed that acadesine and acadesine-based combination interfere with migration in MCL cells. Accordingly, it has been reported that acadesine significantly inhibits the growth of tumors in nude mice xenotransplants of retinoblastoma, by inducing apoptosis and suppressing tumor angiogenesis and macrophage infiltration [33]. Of importance, acadesine and rituximab combination therapy resulted in enhanced cell death in MCL cell lines and primary tumor samples and increased regression of tumor burden in an *in vivo* murine model of MCL.

In summary, our GEP analysis, together with our histological and functional validation studies, suggest that by mainly decreasing the proliferative and survival gene signatures that drive MCL cell growth, the combination strategy associating rituximab to acadesine may represent a new effective therapy to improve MCL patients' outcome and to reduce treatment-associated toxicities.

## METHODS

### Cell lines

Nine human MCL cell lines (GRANTA-519, JVM-2, JEKO-1, Z-138, MAVER-1, REC-1, UPN-1, HBL-2 and MINO) and two CLL cell lines (MEC-1 and MEC-2) were cultured with RPMI 1640, DMEM or IMDM media complemented with 10-20% heat-inactivated fetal bovine serum (FBS), 2 mM L-glutamine, 50 μg/ml penicillin/streptomycin (Life Technologies). Cells were grown in a humidified atmosphere at 37Cº with 5% carbon dioxide and routinely tested for *Mycoplasma* absence by PCR. Additionally, the genetic identity of all cell lines was verified periodically using the AmpFISTR identifier kit (Life Technologies). Genetic characterization of the MCL cell lines is shown in Table 1.

### Primary cultures

Primary cells were obtained from peripheral blood samples of leukemic MCL and CLL patients diagnosed according the WHO criteria. The study was done in accordance with protocols approved by the Ethic Committee of the Hospital Clinic of Barcelona. All patients signed an informed consent according to the Declaration of Helsinki. Biological characteristics of MCL cases are shown in Table 1. Mononuclear cells were isolated by centrifugation on a Ficoll-Hypaque (GE Healthcare) gradient and conserved within the Hematopathology collection of our institution. Cells were either used directly or cryopreserved in liquid nitrogen in the presence of 10% dimethyl sulfoxide, 60% FBS and 30% RPMI 1640. Cells were cultured in a supplemented RPMI medium likewise cell lines.

### Assays of cytotoxicity

MCL cell lines were incubated with acadesine (kindly provided by Advancell) at doses ranging from 0.1 to 2 mM for 24 or 48 hours. Inhibition of proliferation was measured using the MTT assay and the IC_50_ was defined as the concentration of drug required to reduce cell proliferation by 50%. Cell viability was quantified after dual staining of cells with annexin V-fluorescein isothiocyanate (FITC)-conjugated and propidium iodide (PI) (Bender Medsystems), followed by flow cytometry analysis on a FACScan flow cytometer using CellQuest and Paint-A-Gate softwares (Becton Dickinson). Lethal dose 50 (LD_50_) was defined as the concentration of drug required to reduce cell viability by 50%.

Primary MCL cells were incubated with acadesine at two different doses (1 and 2 mM) for 24 hours. In these samples, the viability of tumoral B (CD19+) and accompanying T (CD3+) cells was simultaneously analyzed by triple labeling of the samples with anti-CD19-phycoerythrin (PE) and anti-CD3-FITC antibodies (Becton Dickinson), and allophycocyanin (APC)-conjugated annexin V (Bender Medsytems) on a FACScalibur flow cytometer.

For bendamustine and bortezomib combinations, MCL lines were incubated simultaneously with acadesine (0.5 and 1 mM), and bortezomib (2.5 and 5 nM) or bendamustine (25 and 50 μM) for 48 hours. Cytotoxicity was analyzed by the MTT assay. Combination indexes (CI) were analyzed with Calcusyn software (Biosoft), based in the Chou & Talalay's method [46]. Drug combinations with a CI lower than 1.0 were considered as synergistic.

For acadesine-rituximab studies, cell lines were pre-incubated for 24 hours with acadesine (0.5 and 1 mM for MCL cells and CLL cell lines, 0.1 and 0.25 for CLL primary cases), followed by a 24 hour exposure to 20 and 40 μg/ml of rituximab (1 and 2 μg/ml for JEKO-1 cells). Because rituximab induces cytotoxicity in part by a complement-mediated mechanism [39], we added 10% of human AB plasma together with rituximab as a source of complement proteins. Cytotoxicity and CI values were quantified as above. When indicated, cells were exposed to 1 μg/ml of recombinant human soluble CD40L (rhsCD40L, Sigma) and 20 ng/ml IL-4 (R&D Systems) 1 h before acadesine exposure.

### Xenograft mouse model

Female 6 to 8-week-old CB17-severe combined inmunodeficiency (SCID) mice (Charles River Laboratories Inc) were bred under pathogen-free conditions at the animal facility of our institution using a protocol approved by the Animal Testing Ethical Committee of the University of Barcelona (CEAA).

Mice were inoculated subcutaneously in the right flank with 1x10^7^ JEKO-1 cells in Matrigel™ Basement Membrane Matrix (1:1) (Becton Dickinson). When tumors were palpable and reached a volume of approximately 200 mm^3^, animals were randomly assigned into different groups with 6 mice per cohort and treated with acadesine at dose level of 400 mg/kg body weight five days a week, rituximab at 10 mg/kg body weight once a week, both drugs as above or the equivalent volume of vehicle, for 18 days. The shortest and longest diameters of the tumor were measured with external calipers twice a week. Tumor volume was calculated using the following standard formula: (the shortest diameter)^2^ x (the longest diameter) x 0.5. Mice were sacrificed at the end of the treatment according to institutional guidelines. Harvested tumors were snap-frozen in Tissue-Tek® O.C.T. medium (Sakura Tissue Tek) or formalin-fixed before paraffin embedding on silane-coated slides in a fully automated immunostainer (Bond Max; Vision Biosystems).

### Gene expression studies

Total RNA was extracted from 3 representative harvested tumors of each group, using the RNeasy Mini Kit (Qiagen). RNA quality and quantity was determined using a 2100 Bioanalyzer (Agilent Technologies) and only high quality RNA samples were processed to Affymetrix GeneChip HT HG-U219 arrays. Sample preparation for microarray hybridization was performed following Affymetrix's protocols. Briefly, from 150 ng of total RNA, a biotin labeled cRNA was generated by reverse transcription. Following cRNA fragmentation, the sample was hybridized on the GeneChip HT HG-U219 perfect-match-only (PM) Array Plate. After hybridization and washes, scanning was processed in the Gene Titan instrument, a fully automated array system. The analysis of the scanned images and the determination of the signal value for each probe set of the array were obtained with GeneChip® Command Console® Software (AGCC) (Affymetrix). Raw data were normalized using the Robust Multichip Analysis (RMA) algorithm of the BioConductor Affy Package.

Differential expression data analysis was carried out using the Multiexperiment Viewer Platform (TM4-MEV) [47]. To compare each treatment to the control, we determined the number of statistically significant up- and down-regulated genes using Rank Products methodology [48] setting up a false discovery rate (FDR) below 0.2 and an absolute fold change (FC) above 1.4.

Gene function was assigned based on Database for Annotation, Visualization and Integrated Discovery (DAVID) tool (http://www.david.abcc.ncifcrf.gov) and Gene Ontology (http://www.geneontology.org) in terms of biological process and molecular function. Gene sets with a P value below 0.05 were considered as significant. Primary microarray data are available at the Gene Expression Omnibus (GEO) of the National Center for Biotechnology Information (GSE47871).

An enrichment pathway analysis was done using the gene set enrichment analysis (GSEA) desktop application version 2.0 (GSEA, Broad Institute at MIT, Cambridge, MA; http://www.broadinstitute.org/gsea/) in order to find significant gene signatures using experimentally derived custom gene sets. Gene sets were downloaded from http://lymphochip.nih.gov/signaturedb/index.html. A two-class analysis with 1,000 permutations of gene sets and a weighted metric was used. Gene sets with FDR below 0.05 were considered as significant.

### Immunohistochemistry staining

Immunohistochemical staining studies were performed as previously described [49]. The following antibodies were used: anti-metallothionein (Dako) that recognizes the metallothionein isoforms 1 and 2, anti–cleaved caspase-3 (Cell Signaling Technology) and anti–phospho-Histone H3 (Epitomics). Preparations were evaluated with an Olympus DP70 microscope by means of a 40×/0.75 NA objective and DPManager software v2.1.1 (Olympus).

### Chemotaxis studies

JEKO-1 cells (10^7^ cells/mL) were washed twice and serum-starved for 1.5 hours in FBS-free RPMI. Acadesine 1 mM and rituximab 2 μg/ml were added simultaneously for 3 additional hours, and cells were diluted to 5 × 10^6^ cells/mL with 0.5% bovine serum albumin (BSA; Sigma) in RPMI. One hundred microliters (5 × 10^5^ cells) was added to the top chamber of a Transwell culture polycarbonate insert with 6.5-mm diameter and 5 μm of pore size (Corning). Inserts had been previously transferred to wells containing 600 μL of RPMI with 200 ng/mL of human recombinant CXCL12 (Peprotech). After 3 hours of incubation at 37°C, 100 μL was collected in triplicate from each lower chamber and counted by flow cytometry (Attune, Life technologies) for 12 seconds under constant flow rate. Migration is represented as the number of CXCL12-stimulated migrating cells.

### Statistical analysis

All statistical analysis were performed using GraphPad Prism 4.0 Software (Graphpad Software). Comparison of means between two groups of samples was evaluated by non-parametric Mann-Whitney test. Results were considered statistically significant when *P*≤ 0.05 (*P<0.05, **P<0.01, ***P<0.001).

## SUPPLEMENTARY TABLE


